# Three verbal fluency tasks: Normative data and convergent validity in Argentines over 50 years

**DOI:** 10.1590/1980-5764-dn-2022-0026

**Published:** 2022-11-04

**Authors:** Pablo Martino, Mauricio Cervigni, Nelson Portillo, Miguel Gallegos, Daniel Politis, Miguel Ángel De Bortoli, Jorge Vivas

**Affiliations:** 1 Universidad Nacional de Rosario, Facultad de Psicología, Centro de Investigación en Neurociencias, Rosario, Argentina.; 2 Consejo Nacional de Investigaciones Científicas y Técnicas, Buenos Aires, Argentina.; 3 Universidad Católica del Maule, Facultad de Ciencias de la Salud, Talca, Maule, Chile.; 4 Pontificia Universidade Católica de Minas Gerais, Programa de Pós-Graduação em Psicología, Belo Horizonte MG, Brazil.; 5 Universidad de Buenos Aires, Facultad de Psicología, Cátedra de Neuropsicología, Buenos Aires, Argentina.; 6 Universidad Nacional de San Luis, Facultad de Psicología, PROICO Salud Humana, Enfoque Integrado Psicobiológico, San Luis, Argentina.; 7 Universidad Nacional de Mar del Plata, Facultad de Psicología, Instituto de Psicología Básica, Aplicada y Tecnología, Mar del Plata, Argentina.

**Keywords:** Neuropsychological Tests, Language, Speech, Argentina, Reproducibility of Results, Verbal Fluency, Testes Neuropsicológicos, Idioma, Fala, Argentina, Reprodutibilidade dos Testes, Fluência Verbal

## Abstract

Verbal fluency tasks are frequently used in neuropsychological assessment, standing out for their easy application and good sensitivity to early cognitive impairment. However, in Argentina, the availability of updated norms is limited, especially for the action fluency variant. There is also little evidence of validity.

**Objectives:**

The aim of this study was to obtain Argentine norms for three verbal fluency tasks and to analyze their convergent validity.

**Methods:**

Using a nonprobability sampling method, 303 Argentines from a nonclinical population (age mean=66.8, 50–91 years) were recruited to participate in this study. Those with medical conditions that could compromise neuropsychological performance were excluded. Three verbal fluency tasks (i.e., phonological, semantic, and action), the Montreal Cognitive Assessment (MoCA) test, and the Digit Span-WAIS III test were administered. Correlations and multiple regressions were subsequently performed.

**Results:**

Education and age significantly explained 11.8% of the variance in phonological fluency, 15.8% of the variance in semantic fluency, and 20.2% of the variance in action fluency. Hence, the normative data varied according to educational level and age group, with normal performance limit values between 9 and 14 for phonological fluency, 11 and 18 for semantic fluency, and 8 and 17 for action fluency. Positive correlations were obtained between all verbal fluency tasks, as well as between the MoCA test and the Digit Span test.

**Conclusions:**

This study supports the applicability of three verbal fluency tasks in an Argentine context by providing age- and education-corrected norms and acceptable evidence of convergent validity.

## RESUMO

As tarefas de fluência verbal são frequentemente utilizadas na avaliação neuropsicológica, destacando-se pela facilidade de aplicação e boa sensibilidade ao comprometimento cognitivo incipiente. No entanto, na Argentina, a disponibilidade de padrões atualizados é limitada, especialmente pela variante de fluência de ações. Assim sendo, a evidência de validade é escassa.

**Objetivos:**

Obter normas argentinas para três tarefas de fluência verbal e analisar sua validade convergente.

**Métodos:**

Usando um método de amostragem não probabilística, 303 argentinos de uma população não clínica (idade M=66,8, 50–91 anos) foram recrutados para participar deste estudo. Foram excluídos aqueles com condições médicas que pudessem comprometer o desempenho neuropsicológico. Três tarefas de fluência verbal (ou seja, fonológica, semântica e ações), o teste *Montreal Cognitive Assessment* (MoCA) e o teste Digit Span-WAIS III foram administrados. Correlações e regressão múltipla foram realizadas posteriormente.

**Resultados:**

A escolaridade e a idade explicaram significativamente 11,8% da variância da fluência fonológica, 15,8% da variância da fluência semântica e 20,2% da variância da fluência de ações. Assim, os dados normativos variaram de acordo com a escolaridade e a faixa etária, com limite de desempenho normal entre 9 e 14 para fluência fonológica, 11 e 18 para fluência semântica e 8 e 17 para fluência de ações. Foram obtidas correlações positivas entre todas as tarefas de fluência verbal, bem como entre estas, o teste MoCA e o *Digit Span*.

**Conclusões:**

Este estudo apoia a aplicabilidade de três tarefas de fluência verbal no contexto argentino, fornecendo normas corrigidas por idade e educação e evidências aceitáveis de validade convergente.

## INTRODUCTION

Neuropsychological evaluation (NPE) is the test of choice to characterize the state of higher brain functions, such as attention, memory, language, agnosias, or executive functions^1^. Regarding the use of NPE among adults, this type of procedure offers useful information to clinical neurologists to confirm or rule out the presence of amnesias, aphasias, hemineglect, agnosias, or other neuropsychological syndromes. It also constitutes a key tool in the early detection of cognitive impairment and dementia, all growing problems that are of great concern to public health systems in the Americas and the world^2,3,4^.

Although NPE is highly variable in terms of strategies and instruments, since it depends mainly on the patient’s problems^5,6^, most evaluations usually contain specific language tests, covering one or several linguistic domains, such as verbal fluency (VF), comprehension, repetition, or naming. Among the most commonly used language tests, VF tasks stand out for their easy and brief application^7^ and for their good sensitivity to early cognitive impairment and other neurological pathologies^8^. VF is an appropriate strategy for assessing healthy and cognitively impaired older adults with low educational level^9^. Verbal fluency is defined as the ability to produce spontaneous speech, without excessive pauses or word search failures^10^ and requires verbal information retrieval strategies, with simultaneous activity of several cognitive processes, such as sustained attention, semantic memory, working memory, processing speed, flexibility, and even inhibitory mechanisms^5^.

From a procedural point of view, VF tasks require the examinee to say as many words as possible in a given time, usually 1 min^5,11^. Its main variants are two, phonological fluency, in which words beginning with a specific letter must be mentioned, avoiding proper nouns, conjugations of the same verb, or words of the same family, and semantic fluency, which requests words belonging to a specific category (e.g., animals). A less conventional variant that has recently begun to be used is the action VF task^12^, in which the examinee is asked to enunciate verbs in Spanish ending in ar, er, or ir.

All neuropsychological tests require norms or scales according to their correct interpretation; that is, reference values that allow the professional to contrast the scores of the person examined with the scores of the general population. In this regard, the literature on the subject warns about the influence of age and education on the performance of neuropsychological tests^1,8^ and, therefore, it is advisable to take into account these demographic variables in the construction of norms. It is important to emphasize that the norms should come from the same cultural context in which the administration of the test is planned, since the performance of a test may vary significantly among subjects of different nationalities^13,14^. Furthermore, according to the recommendations of the Argentine Association for Study and Research in Psychodiagnostics^15^, it is necessary to use updated norms that are less than 10 years old. Regarding VF task norms in Argentina, a recent systematic review^16^ reported nine normative studies^7,8,10,17,18,19,20,21,22^. Most of these normative studies are more than 10 years old and focus only on the two classical variants of VF (i.e., phonological or semantic), with the exception of Abraham and collaborators^17^ who provide norms for the action VF task.

In addition, as with any psychometric test, it is essential that neuropsychological tests are valid and reliable^23,24^ and that the analysis of their metrics takes into account different cultural contexts, since a neuropsychological test may work correctly in one context, but not in another. Regarding VF tasks, in general, acceptable metric properties are reported^11,12,25^, although in Argentina, as Martino et al.^16^ have warned, the volume of psychometric studies is limited. In fact, there is only one study with an adult population^18^ in which the validity of the semantic VF task was tested. Results showed significant correlations with the Trail-Making Test, part A (r=-0.36) and part B (r=-0.40), a test that evaluates attention and flexibility, respectively, and with the Porteus Maze test (r=0.26) that evaluates mental planning. Fernández et al.^18^ proposed that prefrontal cortex activity is the common factor that would group the measures of these three instruments.

The present study has two objectives: first, to obtain updated normative data for three variants of VF tasks in Argentine adults; and second, to analyze whether these tasks present adequate convergent construct validity. The information provided in this study will enrich the process of neuropsychological assessment and diagnosis in Argentina.

## METHODS

### Study type

The present study falls within the field of psychometric research or instrument testing based on the classification devised by Ato et al.^26^

### Sample

**Figure 1.**
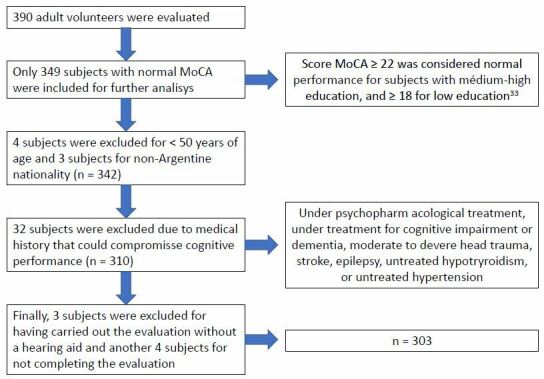
Participant selection criteria and process.

A total of 303 Argentines, aged 50 years or more, participated in the study. Figure 1 reports the sample selection criteria. For this study, nonprobabilistic, purposive sampling was carried out between November 8 and 26, 2021, in the city of Rosario, Argentina, as part of a community campaign for the prevention of cognitive impairment and dementia.

The mean age of the sample was 66.8 years (SD=9.2, min.=50, max.=91) and 74.9% were female. A total of 57.4% reported >12 years of education (mean=18.1, SD=3.4), 34.7% between 8 and 12 years of education (mean=11.6, SD=1.1), and 7.9% reported ≤7 years of education (mean=6, SD=1.4).

### Instruments

#### Verbal fluency tasks^5,11^

Phonological, semantic, and action VF tasks were administered. Regarding phonological VF, participants were asked to name words beginning with the letter “p,” with the exception of proper nouns, verb conjugations, and words of the same family. The semantic VF was evaluated by asking participants to name as many animals as possible. For the action variant, infinitive verbs in any of their endings (i.e., ar, er, or ir) were requested. For each task, participants were given 60 s, and one point was awarded for each correct word. A higher score was indicative of a higher VF. Previous studies showed good psychometric properties^11,12,16,25^.

#### Montreal Cognitive Assessment (MoCA)^27^

It is a screening test widely used for the global assessment of neuropsychological performance in adulthood. It is characterized by its easy application, between 10 and 15 min, and is composed of tasks that demand visuospatial and visuoconstructive skills, executive functions, memory, attention, calculation, language, and temporospatial orientation. The maximum score is 30 points, and a higher score is indicative of better performance. This test has been translated into Spanish by the same authors of the original version with acceptable validity and reliability indicators^28,29,30^, including validation and norm studies in the Argentine population^31,32,33^.

#### Digit Span-WAIS III test^34^

The test consists of two parts, namely, forward and backward, which evaluates attentional span and working memory, respectively. The “forward” part requires the repetition of a list of numbers in the same direction as they were formulated by the evaluator, while the “backward” part requires repeating the numbers in the opposite order. One point is obtained for each correct attempt with a total of 16 points forward, 14 points backward, and 30 points between both parts. Higher scores indicate greater attentional span/working memory. Good psychometric properties were reported^34^.

### Data collection procedure

The evaluation of the participants was carried out in the context of a prevention campaign carried out in November 2021, in Rosario, Argentina. The campaign was a university extension activity organized and coordinated by the researchers themselves. Its purpose was to raise awareness of protective habits of cognitive functions and routinely assess the general cognitive status of interested adult bystanders. The activity had the support and logistics of the state university of that same town, with tents installed in public spaces and other material means. After receiving brochures on protective habits and being evaluated with the free routine cognitive test (MoCA), the bystanders were invited to participate in the research, signing their consent and subsequent resolution of the specific neuropsychological tests (Digit Span-WAIS III test and three VF tasks). The instruments were administered by researchers and professionals in collaboration with a group of advanced psychology students, who were systematically trained through theoretical and practical workshops.

### Ethical and legal aspects

The research was conducted in accordance with the Declaration of Helsinki. Only subjects who agreed to participate voluntarily and gave written informed consent were included in the study. The study was approved by the Research Ethics Committee of the School of Psychology of the National University of Rosario, Argentina.

### Data analysis

The SPSS v26.0^©^ was used. Measures of central tendency and dispersion were obtained for the VF tasks, total MoCA, and the three Digit Span test scores (i.e., total, forward, and backward). Bivariate correlations were performed to analyze the association of years of education and age with VF task performance. Then, a stepwise multiple regression analysis was performed to estimate the extent to which years of education and age explain performance on VF tasks. For this purpose, years of education and age were entered as predictor variables, and phonological, semantic, and action VF scores were considered explained variables.

Regarding the normative data, normal performance limit values were established based on the SD of the mean. According to *DSM-V* ^35^, values below this limit are considered indicative of poor performance.

Later, to analyze the convergent construct validity, a possible correlation was tested between the three VF tasks and between each of these and the MoCA and Digit Span test scores. It is important to note that while the MoCA test provides a measure of general neuropsychological performance, the Digit Span test provides measures of attentional span and working memory, all of which are theoretically constructs related to VF. Consequently, positive correlations should be obtained between VF tasks, the MoCA test, and the Digit Span test.

To determine the use of parametric or nonparametric methods, the normal distribution (Kolmogorov-Smirnov) was tested. In the absence of normality, correlations were performed according to Spearman’s coefficient. For all analyses, a value of p<0.05 was considered statistically significant.

## RESULTS

Descriptive statistics were obtained for phonological, semantic, and action fluency tasks and for the remaining neuropsychological assessments (Table 1).

**Table 1.** Descriptive of three verbal fluency tasks, MoCA test, and Digit Span test (n=303).

Neuropsychological tests	Mean (SD)
VF phonological	17.39 (5.5)
VF semantic	20.85 (5.9)
VF action	19.96 (7.5)
MoCA total	24.27 (3.2)
DigTest total	14.76 (3.9)
DigTest forward	9 (2.3)
DigTest backward	5.76 (2)

VF: verbal fluency; DigTest: Digit Span test; SD: standard deviation.

Influence of age and education on VF tasks

As shown in Table 2, the three VF tasks correlated positively with years of education and negatively with age.

**Table 2.** Bivariate correlations.

	VF phonological	FV semantic	FV action
VF phonological	–	0.506*	0.586*
VF semantic	–	–	0.599*
Years of education	0.262*	0.232*	0.443*
Age	-0.257*	-0.347*	-0.199*
MoCA total	0.415*	0.378*	0.439*
DigTest total	0.399*	0.368*	0.509*
DigTest forward	0.245*	0.318*	0.430*
DigTest backward	0.392*	0.344*	0.490*

VF: verbal fluency; DigTest: Digit Span test; s: Spearman. *Correlation is significant at the <0.01 level (two-tailed).

### Multiple regression models

Regression models indicate that education and age significantly explained 11.8% of the variance of phonological VF (r^2^ corrected=0.118, F=21.27, p<0.01), 15.3% of the variance of semantic VF (r^2^ corrected=0.153, F=28.3, p<0.01), and 20.2 % of the variance of action VF (r^2^ corrected=0.202, F=38.15, p<0.01). Likewise, for each new year of education, the phonological VF score increases by 0.28 units (β=0.282; t=4.5, p<0.01), 0.26 units for the semantic VF (β=0.263; t=3.96, p<0.01), and 0.65 units for the action VF (β=0.658; t=8.09, p<0.01). In contrast, the phonological VF score decreases 0.14 units for each new year of age (β=−0.145; t=4.47, p<0.01), 0.21 units for the semantic VF (β=−0.212; t=6.17, p<0.01), and 0.13 units for the action VF (β=−0.132; t=3.13, p<0.01).

### Convergent validity

As shown in Table 2, VF tasks correlated positively with each other, as well as with total MoCA and all Digit Span test scores (i.e., total, forward, and backward).

## DISCUSSION

The present study focused on three VF tasks with the purpose of obtaining norms adjusted to the Argentine context and analyzing convergent validity. For this purpose, 303 Argentines over 50 years of age from a nonclinical population were evaluated with three VF tasks (i.e., phonological, semantic, and action), the MoCA test, and the Digit Span test.

The finding to highlight is the influence of education and age on the performance of all VF tasks. These results are consistent with a large body of scientific literature that has reported the modulating effect of these demographic variables on the execution of VF tasks and neuropsychological tests in general^1,8^. In this regard, our regression analyses confirm that increasing education increases VF in all its variants and that, on the contrary, increasing age significantly reduces fluency. Based on the impact of education and age on the performance of VF tasks, Argentine norms adjusted for education and age range were developed (Table 3). These new norms are preceded by nine other Argentine normative studies of the adult population^7,8,10,17–22^, and according to our review of the available literature, it is the second study with normative data for the VF variant of action, complementing the work of Abraham et al.^17^.

**Table 3.** Normative data for three verbal fluency tasks in Argentines over 50 years old according to age and education (n=303).

	Age		Education		
			≤7 years	8–12 years	>12 years
Phonological fluency	50–59	Mean (SD)	15 (4.6)	17 (5)	19.7 (5.8)
		Normal performance limit	*	12	14
	60–69	Mean (SD)	15 (6.6)	16.2 (6)	19.7 (5.6)
		Normal performance limit	*	10	14
	70–79	Mean (SD)	12 (3.7)	16.4 (6)	17.3 (4.1)
		Normal performance limit	*	10	13
	≥80	Mean (SD)	12.6 (6.8)	12.8 (3.7)	15.7 (5.2)
		Normal performance limit	*	9	10
Semantic fluency	50–59	Mean (SD)	19.2 (6.1)	22.1 (6.5)	23.3 (5.5)
		Normal performance limit	*	16	18
	60–69	Mean (SD)	18 (3.8)	19.2 (5.6)	23.6 (5.9)
		Normal performance limit	*	14	17
	70–79	Mean (SD)	18 (4.7)	20 (5.1)	19.9 (5.1)
		Normal performance limit	*	14	14
	≥80	Mean (SD)	12 (6.2)	14.3 (2.5)	17.5 (5.3)
		Normal performance limit	*	11	12
Action fluency	50–59	Mean (SD)	12.4 (3.9)	17.4 (6.7)	23.5 (6.5)
		Normal performance limit	*	11	17
	60–69	Mean (SD)	12.3 (6.5)	17.9 (6.3)	23.8 (7.6)
		Normal performance limit	*	11	16
	70–79	Mean (SD)	13.8 (7.8)	18 (7.2)	20.7 (6.3)
		Normal performance limit	*	10	14
	≥80	Mean (SD)	11.6 (6.6)	14.2 (6.2)	19.1 (6.1)
		Normal performance limit	*	8	13

The absolute frequency of the number of participants according to age range and educational level is reported: 50–59 years old with ≤7 education, n=7; 8–12 education, n=25; >12 education, n=37; 60–69 years old with ≤7 education, n=6; 8–12 education, n=39; >12 education, n=74; 70–79 years old with ≤7 education, n=8; 8–12 education, n=29; >12 education, n=48; ≥80 years old with ≤7 education, n=3; 8–12 education, n=12; >12 education, n=15; *n smaller than 10 cases, so no normal performance limit value is reported.

Nevertheless, it is important to note the low frequency of subjects with low education. A total of 57.4% had post-secondary studies (>12 years of education), 34.7% had secondary studies (between 8 and 12 years of education), and only 7.9% reported primary education (≤7 years of education).

In turn, the current norms offer normal performance limit values according to education and age, adopting as a criterion a SD from the mean, based on the *DSM-V* criteria for neurocognitive disorders^34^. According to these criteria, scores between 1 and 2 SDs from the mean should be considered indicative of minor neurocognitive disorder (mild cognitive impairment), and more than 2 SDs from the mean, indicative of major neurocognitive disorder (dementia). Due to the low sample size for all subgroups with primary education (n<10 cases), it was considered inappropriate to report normal performance limit values for these subgroups. New studies with a larger sample size will be able to overcome this difficulty.

Despite the value of this type of measurement instrument in neuropsychology, it is important to keep in mind that the isolated administration of any neuropsychological test is insufficient to draw diagnostic conclusions. Hence, it is advisable to carry out evaluations of greater breadth and depth, using multiple neuropsychological tests and the assessment of daily functioning. Other qualitative aspects that may be present in the interview or during the general process of NPE should also be considered. Consequently, two major paradigms or approaches in Clinical Neuropsychological Evaluation are evident: the quantitative of the North American tradition and subject to psychometrics; and the qualitative approach, which advocates a more comprehensive view of the evaluation process, in accordance with the Soviet school and the figure of Alexander Luria^1,23,36^.

Neuropsychological tests do not differ substantially from psychometric instruments used in the field of general psychological assessment and, therefore, require evidence of validity and reliability as well as cultural context adjustment. This study analyzed the convergent validity of three VF tasks, assuming potential associations with other tests assessing close neuropsychological constructs. More precisely, the MoCA test assesses general neuropsychological performance, and the Digit Span test explores attentional and executive processes. Positive correlations were indeed found between the VF tasks themselves and between the MoCA test and the Digit Span test (Table 2). The results of the present study will help to increase the evidence of the construct validity of VF tasks in the Argentine context and to strengthen the volume of local scientific production in neuropsychological assessment.

This study is not without limitations. First, it relied on nonprobabilistic sampling, which compromises the generalizability of the data. Second, the evaluations took place within the framework of a prevention campaign, in tents set up in public spaces. Therefore, the existence of extraneous variables such as disturbing noises or fluctuations in luminosity cannot be ruled out. The researchers are aware that this compromises the internal validity of the study. However, a more ecological setting, such as the one provided by the study, on the contrary, considerably strengthens the external validity of a neuropsychological investigation. Third, the administration of the instruments was carried out by researchers with specific training in neuropsychology and collaborators who were advanced psychology students. The presence of different evaluators could interfere as an extraneous variable depending on the subjectivity of each evaluator. However, it is worth clarifying that all the evaluators participated in workshops and periodic meetings in order to agree on a set of guiding criteria and mitigate potential differences in the administration of the instruments. Future studies should use inter-rater reliability to minimize these differences. Finally, the medical information with which the inclusion-exclusion criteria were defined was based on self-reports provided by the participants, and, thus, omissions or overestimates of disease events could not be ruled out.

This study provides new normative data and acceptable validity evidence for three VF tasks in the Argentine population over 50 years of age. It is recommended to Argentine neuropsychologists to incorporate these tasks into the assessment protocols for adults and older adults.
